# Pro-apoptotic gene BAX is a pan-cancer predictive biomarker for prognosis and immunotherapy efficacy

**DOI:** 10.18632/aging.206003

**Published:** 2024-07-05

**Authors:** Siying Wang, Xuyu Chen, Xiaofei Zhang, Kang Wen, Xin Chen, Jingyao Gu, Juan Li, Zhaoxia Wang

**Affiliations:** 1 Department of Oncology, The Second Affiliated Hospital of Nanjing Medical University, Nanjing 210011, Jiangsu, P.R. China; 2 Department of Gastroenterology, The Affiliated Hospital of Yangzhou University, Yangzhou University, Yangzhou 225012, Jiangsu, P.R. China

**Keywords:** BAX, pan-cancer analysis, immune infiltration, prognosis, TME

## Abstract

Background: Apoptosis Regulator BCL2 Associated X (BAX) is a pro-apoptotic gene. Apoptosis is one of the important components of immune response and immune regulation. However, there is no systematic pan-cancer analysis of BAX.

Methods: Original data of this study were downloaded from TCGA databases and GTEX databases. We conducted the gene expression analysis and survival analysis of BAX in 33 types of cancer via Gene Expression Profiling Interactive Analysis (GEPIA) database. Real-time PCR and immunohistochemistry (IHC) were further performed to examine the BAX expression in cancer cells and tissues. Moreover, the relationship between BAX and immune infiltration and gene alteration was studied by the Tumor Immune Estimation Resource (TIMER) and cBioPortal tools. Protein–protein interaction analysis was performed in the STRING database. Finally, Gene Ontology (GO) and Kyoto Encyclopedia of Genes and Genomes (KEGG) were utilized to evaluate the enrichment analysis.

Results: BAX was highly expressed in most cancers and was associated with poor prognosis in nine cancer types. In addition, BAX showed significant clinical relevance, and the mRNA expression of BAX was also strongly associated with drug sensitivity of many drugs. Furthermore, BAX may participate in proliferation and metastasis of many cancers and was associated with methylation. Importantly, BAX expression was positively correlated with most immune infiltrating cells.

Conclusion: Our findings suggested that BAX can function as an oncogene and may be used as a potential predictive biomarker for prognosis and immunotherapy efficacy of human cancer, which could provide a new approach for cancer therapy.

## INTRODUCTION

In the 21st century, cancer will become the main cause of premature death around the world [1]. It has been proposed that the incidence rate of all cancers will double by 2070 compared to 2020 [2]. Although great progress has been made in the pathogenesis and treatment of cancer, the mortality rate of cancer is still rising [3]. In recent years, with the improvement of public online resources, genomic data is becoming more and more readily available. A pan-cancer analysis of a gene that plays a pivotal role in the occurrence and development of cancer could provide an important theoretical basis for a better understanding of mechanisms in tumor progression.

BAX, (also known as BCL2L4 and Bcl-2-associated X protein) is a pro-apoptotic protein and a member of the Bcl-2 family. It was demonstrated that its full-length is 1664 bp, containing an ORF of 579 bp, a 5′UTR of 64 bp and a 3′UTR of 1021 bp. It was located at chromosome 19q13.33, encoding for a 192 amino acids length polypeptide. The predicted molecular weight is 21.55 kDa and the isoelectric point is 6.75 [4]. The Bcl-2 family could regulate the intrinsic pathways of apoptosis, which is a cell suicide system that is necessary for tumor development and immunity [5, 6]. It has been reported that BAX participates in a critical step of the mitochondria-dependent apoptosis. Because of the stimulation of apoptosis, it was activated at the mitochondrial outer membrane, in order to regulate its permeabilization [7]. It can also be activated in a p53-dependent way through multifarious stimuli [8].

A predisposition to apoptotic death among cells that have acquired a malignant mutation serves to facilitate one of the body’s primary defenses against cancer. Induction of apoptosis is a failsafe and occurs as a result of the tight interweaving of the multiple genes and signaling pathways regulating survival, proliferation, and growth. Interestingly, apoptosis is tightly controlled, and misregulation of apoptosis is a hallmark of human cancers and autoimmune diseases [9]. It is also one of the important components of immune response and immune regulation, which has special significance for immunology [10]. Immune regulation refers to the interaction between immune molecules, immune cells, the immune system and other systems of the body in the immune response, forming a regulatory network of mutual coordination and restriction, so that the body's immune response is at the appropriate level of strength and quality, thereby maintaining the stability of the body's internal environment. Meanwhile, BAX plays a critical role in the interface between innate immunity control and apoptotic signaling [11]. Accumulating studies have indicated that the induction of BAX-regulated apoptosis has become a pivotal strategy for drug resistance and cancer treatment [12].

Importantly, BAX was widely reported due to its remarkable role in tumor progression and therapy. In lung cancer, the Bcl-2/BAX/caspase-3 signaling pathway was shown that it can enhance immunogenicity to inhibit the proliferation of lung cancer cells [13]. Meanwhile, in breast cancer, the degradation of BAX could contribute to its malignancy [14]. Moreover, in colon cancer, the pathway of AKT/Bcl-2/BAX could promote the metastasis of colon cancer cells [15]. Although BAX has a significant impact on the development of multiple cancers, its specific mechanism has not been fully elucidated.

Here, we performed a comprehensive analysis of the BAX gene based on the TCGA dataset, including gene expression and survival analysis, co-expression analysis, gene-set enrichment analysis, immune cell infiltration analysis, gene mutation analysis and so on to estimate the relationship between BAX and tumor progression.

## METHODS

### Gene expression analysis

The BAX gene expression data for 33 types of tumors were extracted from The Cancer Genome Atlas (TCGA) datasets. The online resource of TIMER2.0 was utilized to compare the expression level of BAX between tumor and adjacent normal tissues from a total of thirty-three tumor types [16]. The Human Protein Atlas database (HPA) RNA-seq tissue datasets revealed the RNA expression of BAX in different organs (https://www.proteinatlas.org/) [17].

### Survival prognosis analysis

We logged into the website of GEPIA2.0, which provides the time information and survival information for more than thirty types of cancer to validate the prognostic value of BAX [18]. The function of “Survival Map” module visualizes the prognosis value of BAX, and the cox-PH model was used to calculate the hazards ratio. Then, we downloaded the overall survival (OS) and disease-free survival (DFS) datasets of the gene BAX to obtain the plots below.

### Analysis of BAX expression and clinical relevance

We analyzed the expression of BAX in various cancer stages using box-plot diagrams. The complete information could be downloaded through the GEPIA2.0 datasets. The UALCAN online tool could also calculate the available clinical information such as age, race and gender [19]. The Gene Set Cancer Analysis (GSCA) software suggested a link between drug sensitivity and BAX expression [20]. The pROC package and ggplot2 package of R were used for plots of ROC curve. The data are extracted from TCGA datasets, and expression values were Log2 transformed.

### Genetic alteration analysis

The cBioPortal website (https://www.cbioportal.org) indicated the features of genetic alteration and mutation sites across multiple tumors [21]. The difference can be clearly comprehended by calculating their percentages. Next, we used the GSCA gene set to analyze the relationship between BAX and copy number variation (CNV).

### Immune infiltration analysis

We downloaded the pan-cancer datasets from the UCSC website (http://xenabrower.net) [22], and evaluated the immune scores of six immune cell types (CD4+T-cells, CD8+T-cells, B cells, Dendritic cells, Macrophage, Neutrophil) in each tumor based on BAX expression using the TIMER function.

In order to further validate the role of BAX in the above six immune cell types, TIMER2.0 website provides several algorithms, such as EPIC, QUANTISEQ, MCP-COUNTER and so on. We chose the algorithm of TIMER to obtain the scatter plots of the immune infiltration data, and distinguished the difference between BAX expression in various immune infiltration cell types. Moreover, we used the online resource TISIDB (http://cis.hku.hk/TISIDB) to confirm that these Tumor-infiltrating lymphocytes (TILs) related to BAX expression are also correlated with various tumors, using criteria *p* < 0.05 [23].

### Methylation analysis

The GSCA online tool was used to analyze the relationship between BAX and methylation. The GSCA database not only analyzed the methylation expression of BAX in many human cancers, but also indicated the relationships between BAX methylation and immune infiltration cells in each cancer. The MEXPRESS online tool was used to illustrate the relationship between BAX expression and methylation sites.

### Proliferation and metastasis

To explore whether BAX expression was correlated with the proliferation and metastasis of cancers, we concluded the epithelial–mesenchymal transition (EMT) markers [24] and the classic proliferation markers [25] of these biological processes. Next, we calculated the relationship between BAX and these markers via the “Gene_corr” section of TIMER2.0 database.

### Enrichment analysis of BAX-related gene

The BAX Protein-protein interaction (PPI) network was constructed by the STRING platform (Search tool for the retrieval of interacting genes) [26]. We chose the “Homo sapiens” section, and then, we obtained and analyzed over twenty interactions of BAX. In order to visualize the results, we used version 3.8.2 of the Cytoscape software (http://www.cytoscape.org) [27].

GEPIA2.0 was used for the analysis of the top 200 genes most related to the similar expression of BAX. Then, TIMER2.0 was utilized to evaluate their correlation. By combining the above data, the enrichment analysis of KEGG and GO were plotted by the ggplot2 package and clusterProfiler package of R [28]. FDR < 0.05 was used for the enriched analysis.

### Cell lines

We cultured human liver hepatocellular carcinoma cell lines (HepG2, MHCC97H, and Huh-7), LUAD cell lines (PC9, H1975, and A549), COAD cell lines (HT29 and SW480) and corresponding normal cell lines (L02, HBE16 and NCM-460). These cell lines were derived in our laboratory. Various cells lines were cultured in DMEM or RPMI 1640 containing 10% FBS in an incubator at 37°C, with 5% CO_2_ and relative humidity of 90%. Medium was replaced every 2–3 days.

### RT-qPCR and validation of expression level

The real time quantitative polymerase chain reaction (RT-qPCR) was utilized to compare gene expression in different LIHC, LUAD and COAD cell lines. These cells were collected and added with Trizol reagent. We extracted the corresponding RNA and reverse transcribed into cDNA. The primers for BAX were displayed as follows: “Forward: TTTGCTTCAGGGTTTCATCC; Reverse: CA GTTGAAGTTGCCGTCAGA”. The primers for GAPDH were displayed as follows: Forward: 5′-GAAGGTGAAGG TCGGAGTC-3′. Reverse: 5′-GAAGATGGTGATGGGA TTTC-3′. Subsequently, immunohistochemistry (IHC) images were utilized to illustrate the difference of BAX expression between normal tissues and tumor tissues. Then, we proved the protein expression via Clinical Proteomic Tumor Analysis Consortium (CPTCA) database.

### Availability of data and materials

The datasets generated and/or analyzed during the current study are available in the (TCGA) repository (https://portal.gdc.cancer.gov/). These datasets used and/or analyzed during the current study are available from the corresponding author on reasonable request.

## RESULTS

### Gene expression analysis

**Figure 1:**
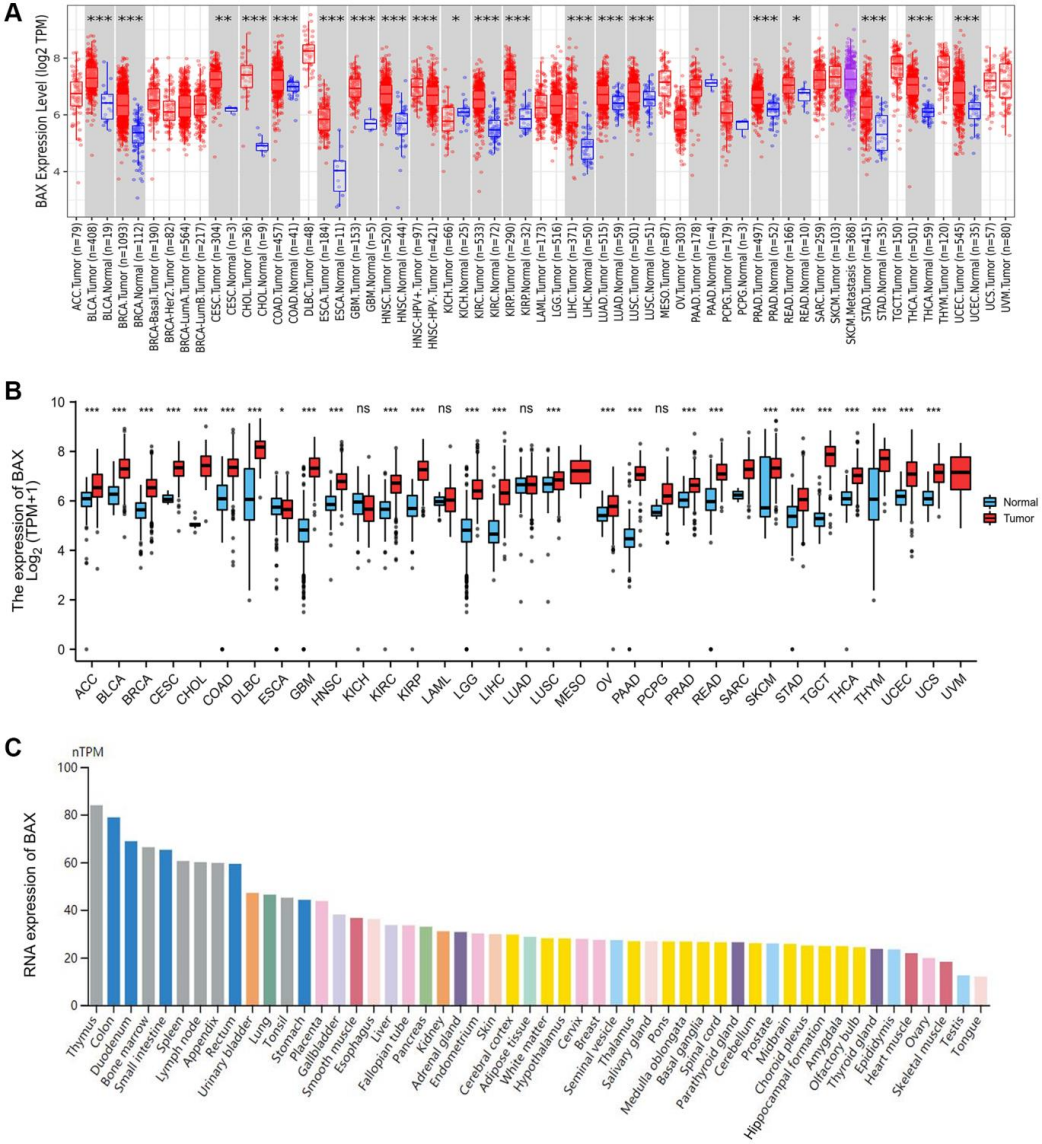
**BAX expression levels in human cancers.** (**A**) The expression of BAX from TIMER2.0 database in different cancer types or cancer subtypes. (^*^p < 0.05; ^**^p < 0.01; ^***^p < 0.001). (**B**) The BAX expression was showed by R package based on TCGA database. (^*^p < 0.05; ^**^p < 0.01; ^***^p < 0.001). (**C**) The RNA expression of BAX in different organs based on HPA dataset.

To explore the expression of BAX across thirty-three tumors, we downloaded relevant data from website TIMER2.0. Compared with normal tissues, the result revealed that the expression level of BAX was highly expressed in eighteen of the twenty-one cancer types for which complete data were available (Figure 1A). In order to supplement the incompleteness of some tumor data, we downloaded more complete gene expression data from TCGA and make a plot using the R package. We found that except for the down-regulated expression of BAX in Esophageal carcinoma (ESCA), BAX was highly expressed in multiple cancer types (Figure 1B and Supplementary Table 1), including Liver hepatocellular carcinoma (LIHC), Brain lower grade glioma (LGG), Glioblastoma multiforme (GBM), Adrenocortical carcinoma (ACC), Kidney renal clear cell carcinoma (KIRC), Kidney renal papillary cell carcinoma (KIRP), Breast invasive carcinoma (BRCA), Bladder urothelial carcinoma (BLCA), Cervical squamous cell carcinoma and endocervical adenocarcinoma (CESC), Cholangiocarcinoma (CHOL), Colon adenocarcinoma (COAD), Lymphoid neoplasm diffuse large B-cell lymphoma (DLBC), Head and Neck squamous cell carcinoma (HNSC), Ovarian serous cystadenocarcinoma (OV), Pancreatic adenocarcinoma (PAAD), Prostate adenocarcinoma (PRAD), Rectum adenocarcinoma (READ), Lung squamous cell carcinoma (LUSC), Skin cutaneous melanoma (SKCM), Stomach adenocarcinoma (STAD), Thyroid carcinoma (THCA), Testicular germ cell tumors (TGCT), Thymoma (THYM), Uterine corpus endometrial carcinoma (UCEC), Uterine carcinosarcoma (UCS) (*P* < 0.001). In addition, the HPA dataset corresponded to mean values of the different individual samples from each tissue, and color-coding is based on each tissue group (Figure 1C and Supplementary Figure 1A–1C). According to the RNA expression level of BAX, we found that BAX was expressed highest in thymus, colon and duodenum tissues.

### Validation of BAX expression

**Figure 2:**
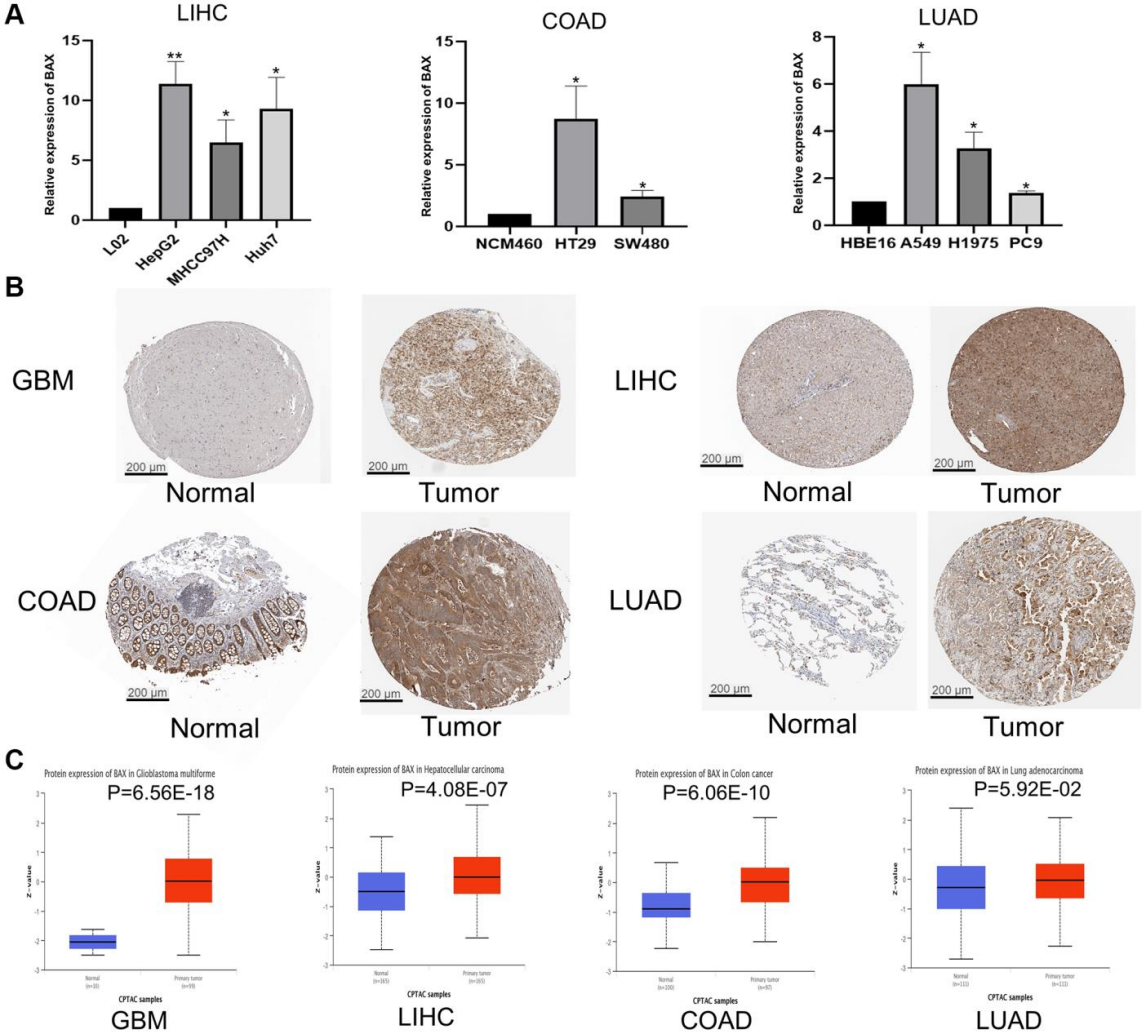
**Validation of BAX in different tissues and cell lines.** (**A**) Bar plot for the relative expression of BAX in LIHC, COAD and LUAD cell lines and corresponding normal L02, NCM460 and HBE16 cell lines, and protein expressions of BAX in LUAD cell lines. (^*^p < 0.05; ^**^p < 0.01). (**B**) Immunohistochemical analysis of BAX in LIHC, COAD, LUAD and GBM tissues and in corresponding normal tissues. (**C**) Protein expression of BAX in LIHC, COAD, LUAD and GBM tissues from CPTAC dataset. (^*^p < 0.05).

Through previous analysis, we found that BAX was highly expressed in LIHC cells and tissues. Therefore, we verified the expression of BAX in LIHC cell lines by RT-qPCR. The expression of BAX was significantly elevated in liver hepatocellular carcinoma cell lines (HepG2, MHCC97H and Huh-7) compared to the normal cell line (L02). We also verified the expression of BAX in LUAD cell lines (PC9, H1975 and A549) and COAD cell lines (HT29 and SW480), compared to the corresponding normal cell lines (HBE16 and NCM-460) (Figure 2A). Moreover, we validated the protein expression of BAX by HPA database (Figure 2B). Deeper staining indicated that BAX expression was higher in tumor tissues than in normal tissues at the protein level. The IHC staining for BAX showed strong staining in GBM, LIHC, COAD and LUAD but weak staining in corresponding normal tissue samples. We analyzed the IHC results provided by the HPA database and compared them with the BAX gene expression data from the CPTAC dataset. As shown in Figure 2C, the results of analysis of data from these two databases were consistent with one another. The CPTAC data also showed that BAX protein expression was higher in some other cancer tissues than in adjacent normal tissues, such as PAAD, OV, KIRC and BRCA (Supplementary Figure 1D).

### Survival prognosis analysis

**Figure 3:**
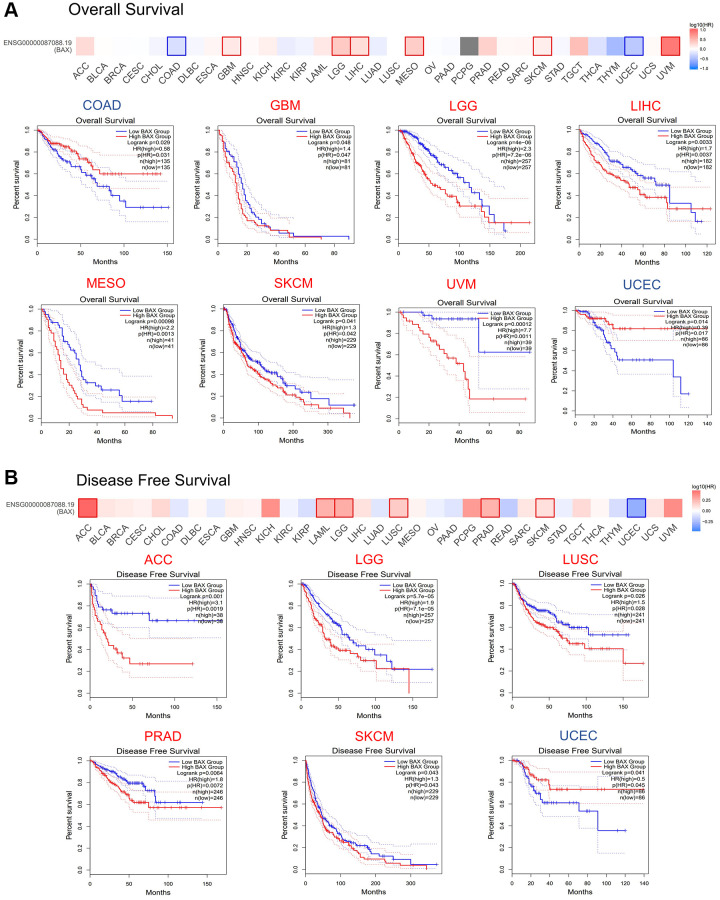
**Prognostic value of BAX in TCGA database.** (**A**) Relationship between BAX expression level and overall survival data in GEPIA2.0. (*P* < 0.05). (**B**) Relationship between BAX expression level and disease-free survival data in GEPIA2.0. (*P* < 0.05).

To reveal the association between BAX expression levels and prognosis in multiple cancer types, we set cut-off high value and cut-off low value to be 50% to distinguish the high-expression group and low-expression group, and axis units was set to months. OS analysis data demonstrated that higher expression of BAX in LGG, SKCM, LIHC, GBM, MESO and UVM (*P* < 0.05) is associated with poorer OS, while lower expression of BAX is associated with COAD and UCEC (*p* < 0.05) (Figure 3A). The data of DFS showed that higher expression in LGG, SKCM, ACC, LUSC, PRAD (*P* < 0.05) is linked with poorer DFS (Figure 3B). However, with higher BAX expression, UCEC patients had longer survival time (*P* < 0.05). In sum, high level of BAX was correlated with poor prognosis in many cancers, and it was a significant high-risk gene in LGG and SKCM because of their poor OS and DFS. Besides, it also suggested that UCEC has a better prognosis.

### Analysis of BAX expression and clinical relevance

**Figure 4:**
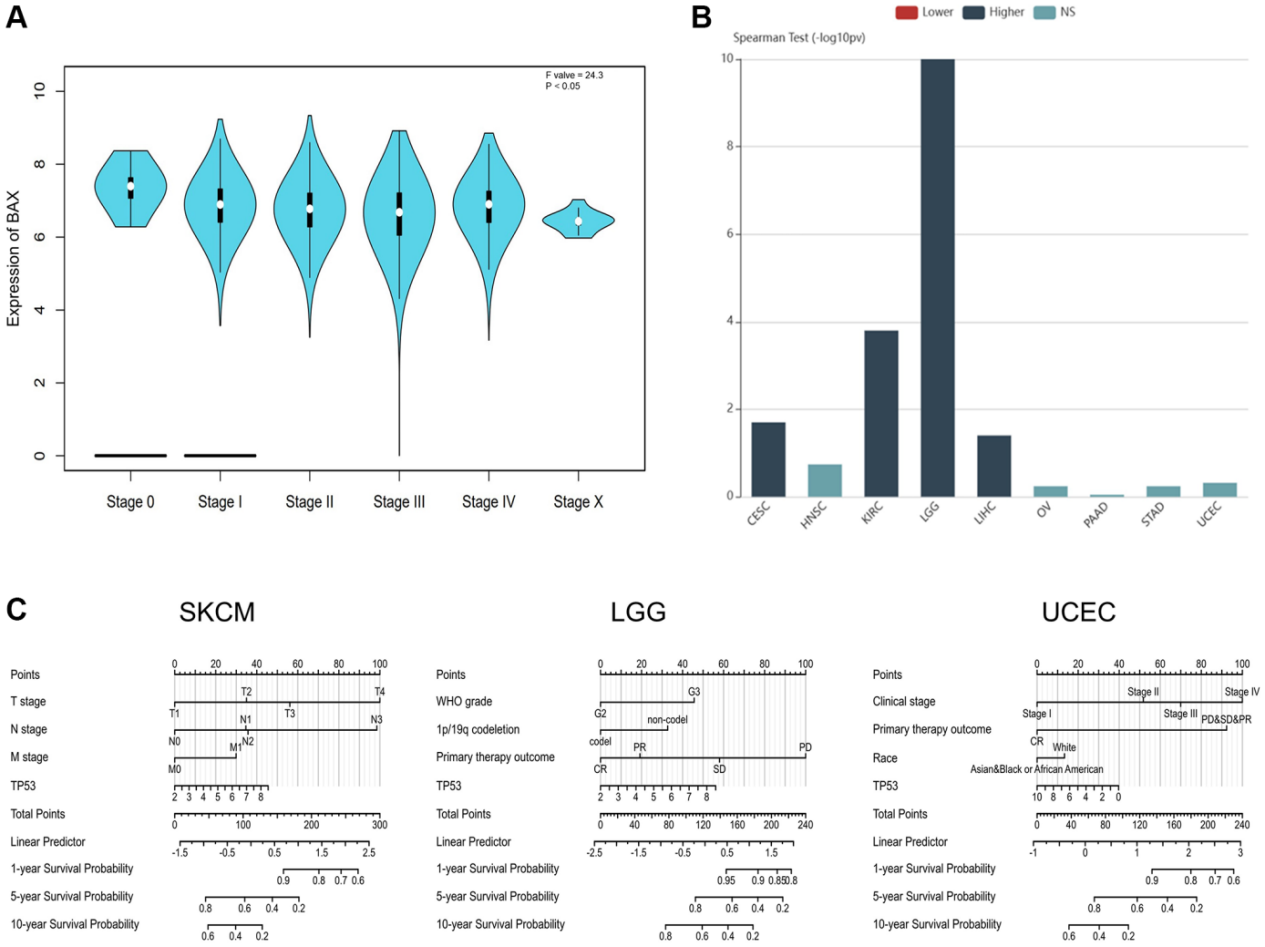
**The correlation between BAX expression and cancer stages.** (**A**) The correlation between BAX expression and pathological stages of 33 TCGA cancers. (*P* < 0.05). (**B**) Correlation between BAX expression and tumor grade in cancers. (*P* < 0.05). (**C**) The expression of BAX in SKCM and UCEC based on individual cancer stages. (*P* < 0.05). (**C**) The nomogram models of SKCM, UCEC and LGG.

**Figure 5:**
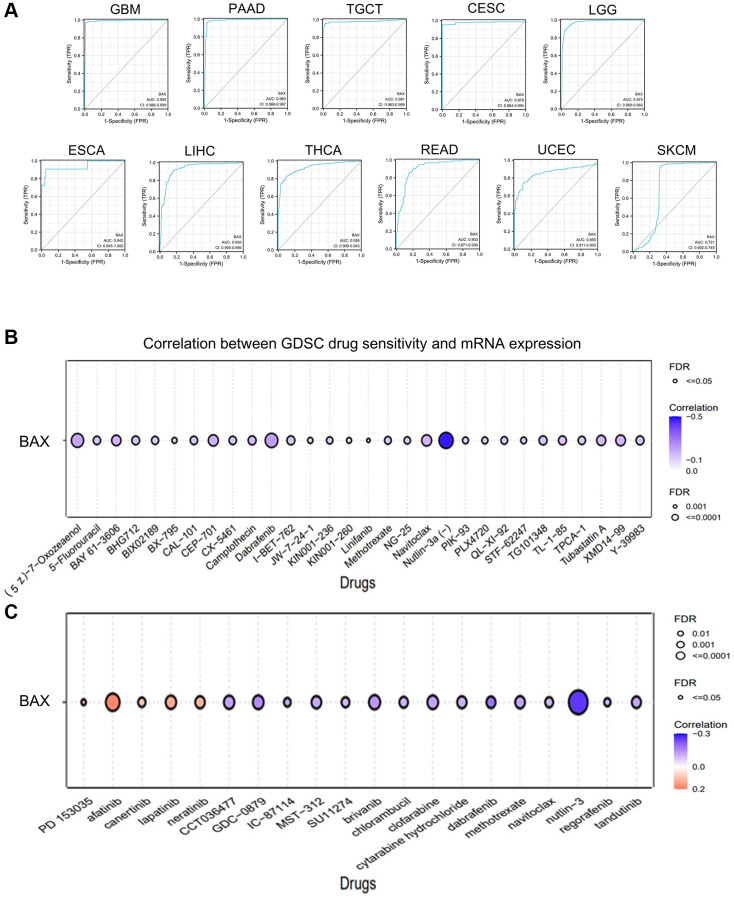
**The correlation between BAX expression and clinical relevance.** (**A**) The ROC curves of BAX in different cancers. (**B**) The correlation between BAX mRNA expression and GDSC drug sensitivity was performed. (**C**) The correlation between BAX mRNA expression and CTRP drug sensitivity was performed.

Subsequently, we examined whether there were relationships between the level of BAX expression and phenotypes. We downloaded the clinical tumor stage data of 33 TCGA tumors from GEPIA2.0 data platform (Figure 4A). The box plot showed that BAX expression was notably correlated with different tumor stages in these cancers (*P* < 0.05). We also found the highest expression of BAX in LGG through tumor grading data in the TISIDB resource library (Figure 4B). We also evaluated the differential expression of BAX in patients with different tumor types of different ages, races and genders (Supplementary Figure 2). We found that BAX expression was significantly associated with age, gender and race in great majority of tumors. Moreover, we established a nomogram model that may predict patients’ survival through R package (Figure 4C). Next, to reveal the clinical sensitivity and specificity, we used the data from TCGA dataset plotting the diagnostic receiver operating characteristic (ROC) for pan-cancer analysis (Figure 5A and Supplementary Figure 3). The results showed that there were many types of cancer whose area under the curve (AUC) value of BAX diagnostic was greater than 0.9, among which GBM was 0.993, TGCT was 0.981, CESC was 0.979, LGG was 0.975, ESCA was 0.942, LIHC was 0.930, THCA was 0.926 and READ was 0.903. In UCEC and SKCM, AUC values were 0.855 and 0.721, respectively. AUC values from 0.8 to 0.9 refer to high performance, and from 0.7 to 0.8 refer to fair performance. In terms of drug sensitivity, we found research showed that drug sensitivity could be associated with mRNA [29]. We sought to find relationships between the mRNA expression of BAX and drug sensitivity by using Genomics of Drug Sensitivity in Cancer (GDSC) and Cancer Therapeutics Response Portal (CTRP) independent drug response datasets (Figure 5B, 5C). In GDSC database, we figured out that the mRNA expression of BAX is strongly associated with (5Z)-7-oxozeaenol, dabrafenib and nutlin-3a (FDR ≤ 0.0001). In CTRP database, the mRNA expression of BAX is significantly correlated with afatinib and nut-lin-3 (FDR ≤ 0.0001). Interestingly, nutlin-3a is the active isoform of nutlin-3, they are small molecule inhibitors that target the MDM2/p53 interaction and are widely used in clinical therapy [30].

### Genetic alteration analysis

**Figure 6:**
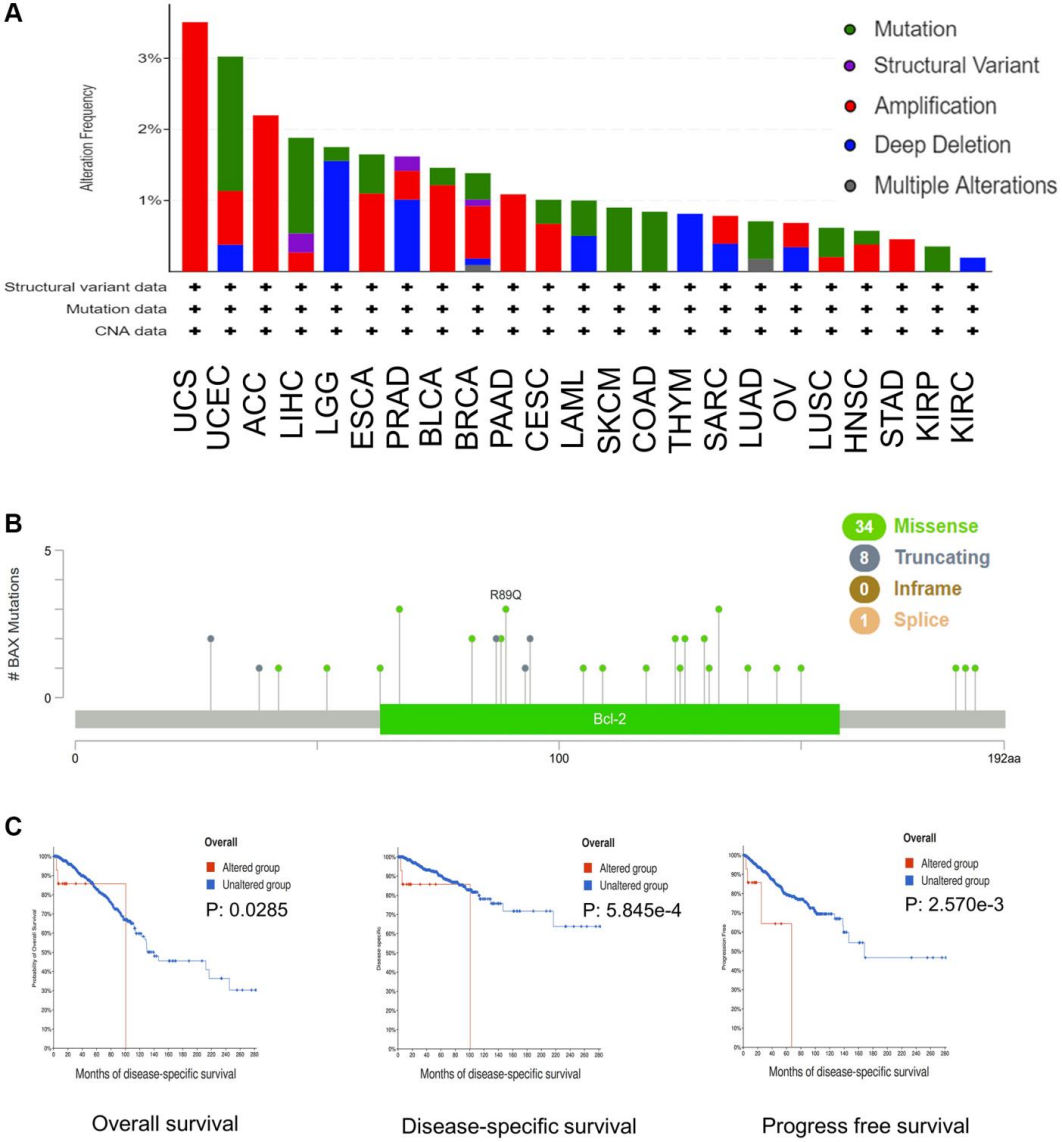
**BAX mutation in different cancer types of TCGA.** (**A**) The alteration frequency with mutation type was performed. (**B**) The mutation sites were performed. (**C**) The potential relationship between mutation status and OS, DSS and PFS of breast cancer. (*p* < 0.05).

Next, we evaluated the mutation status of BAX in various cancer types. The plot proved that the top five cancers with alteration frequency are UCS, UCEC, ACC, LIHC and LGG (Figure 6A). The alteration rate in UCS patients with “amplification” as the primary type was the highest (3.51%, 57 cases). The ‘‘mutation’’ type of copy number alteration was the primary type in UCEC and LIHC patients. And the alteration of copy number in LGG cases was mainly “deep deletion”. In addition, as shown in Figure 6B, the distribution of BAX genetic mutation was evaluated, the main type was missense mutation, and the frequency of R89Q mutation was highest. Subsequently, we found the potential relationship between BAX gene alteration and clinical survival in diverse cancer types. For example, in breast cancer, patients with BAX alteration showed poor prognosis in OS (Overall survival), DSS (Disease-specific survival) and PFS (Progress free survival) (*p* < 0.05) (Figure 6C).

### Analysis of BAX CNV

**Figure 7:**
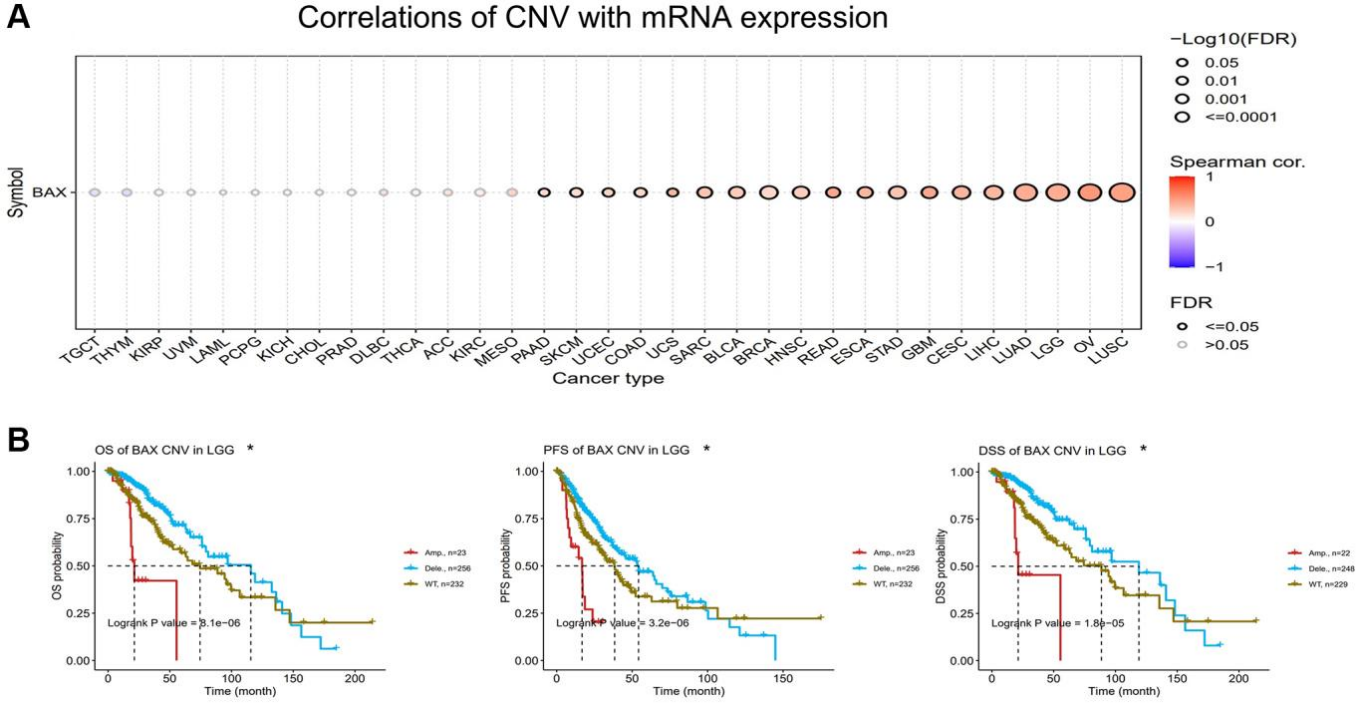
**Analysis of BAX CNV in pan-cancer.** (**A**) A Spearman association between BAX CNV and mRNA was displayed in pan-cancer. (**B**) The potential relationship between BAX CNV and OS, DSS and PFS of LGG. (p < 0.05).

A Spearman correlation between BAX CNV and mRNA was also explored in pan-cancer. There is a remarkable positive correlation between BAX CNV and mRNA expression in LUSC, OV, LGG and LUAD (FDR < 0.0001). It is also positively correlated with LIHC, CESC, GBM, STAD, ESCA, HNSC, BRCA, BLCA, READ, SARC (FDR < 0.01), and UCS, COAD, UCEC, SKCM, PAAD (FDR < 0.05). However, this connection was not notable in MESO, KIRC, ACC, THCA, DLBC, PRAD, CHOL, KICH, PCPG, LAML, UVM, KIRP, THYM and TGCT (Figure 7A). In order to further study the effect of BAX CNV on prognosis, we mainly evaluated LGG, which has a significant positive correlation with it (FDR < 0.0001). The alteration type of LGG is mainly “deep deletion” alteration. Interestingly, cases with “deep deletion” alteration showed better prognosis in OS, DSS and PFS (*P* < 0.05) (Figure 7B).

### Immune infiltration analysis

**Figure 8:**
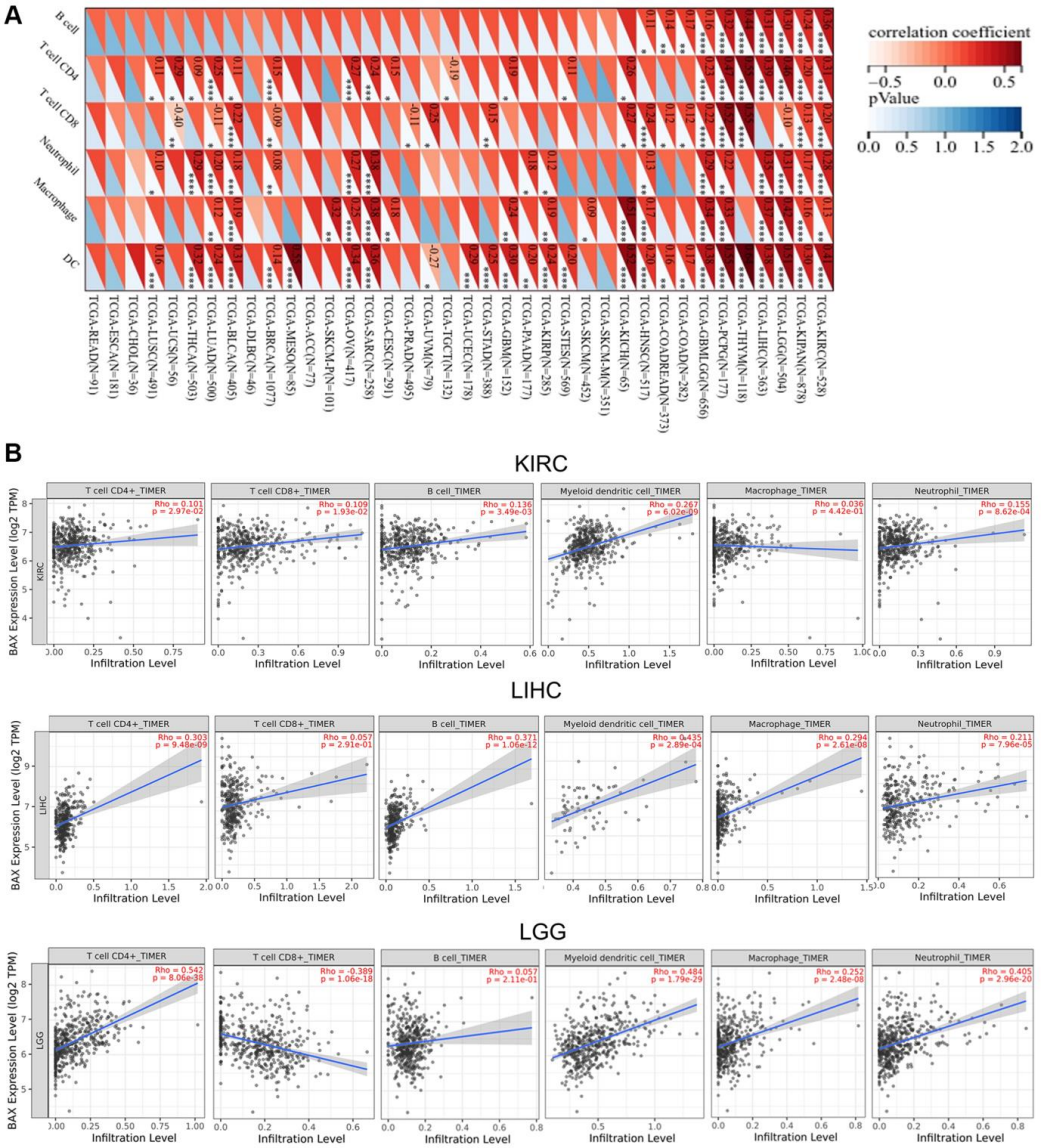
**Correlation analysis between BAX expression and the expression degree of immune cells obtained from TCGA.** (**A**) The correlation of BAX expression with six types of immune cells (B cell, CD4+ T cell, CD8+ T cell, neutrophil, macrophage, and myeloid dendritic cell) in pan-cancer. (**B**) The top three tumors with the highest correlation between the degree of immune infiltration and BAX expression were KIRC, LGG and LIHC via TIMER algorithm. (*P* < 0.05).

**Figure 9:**
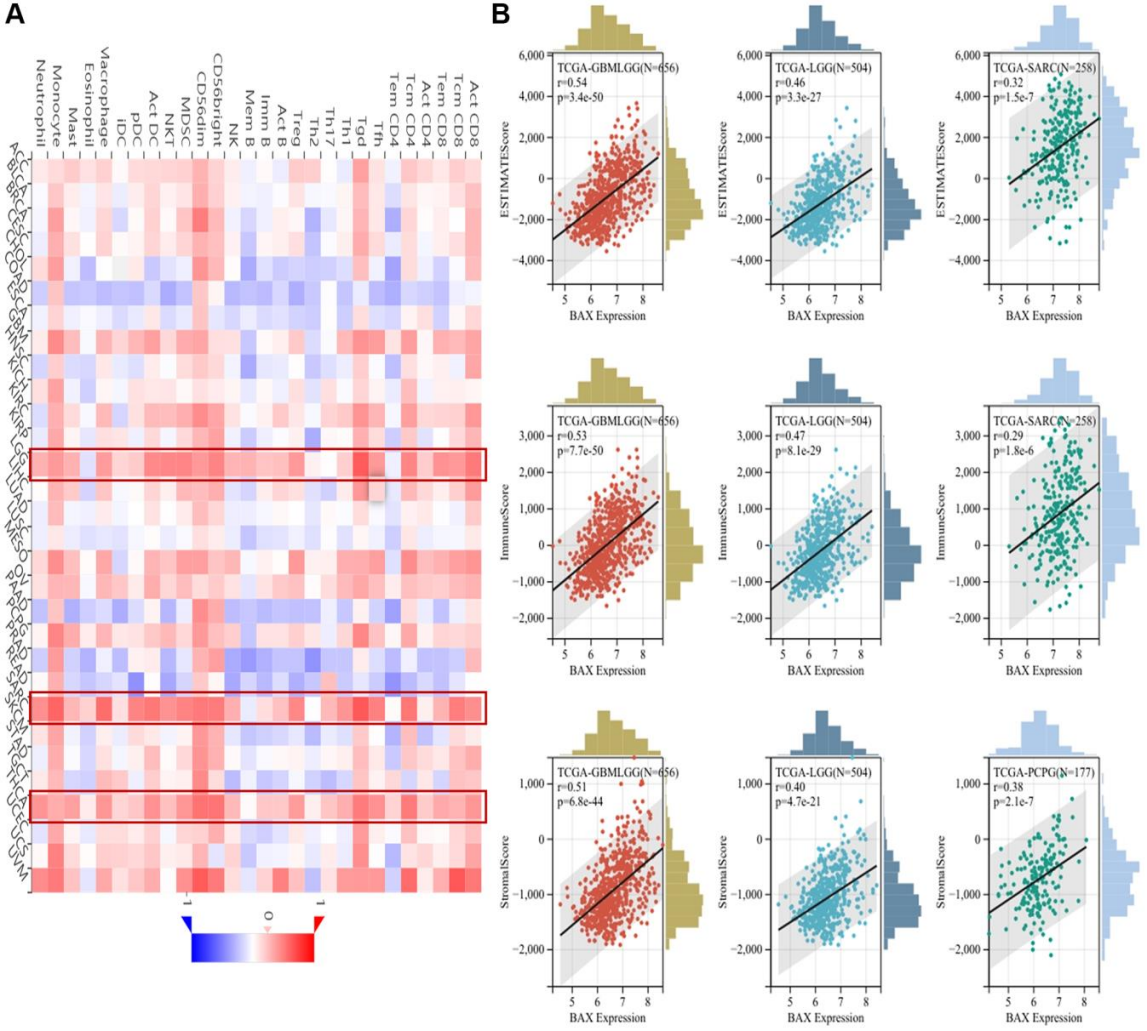
**Correlation analysis between BAX expression and the abundance of immune infiltration.** (**A**) Correlation analysis between abundance of tumor-infiltrating lymphocytes and BAX expression in different cancer types. (**B**) The top three cancers were GBMLGG, LGG and SARC (ESTIMATEScore); GBMLGG, LGG and SARC (ImmuneScore); GBMLGG, LGG and PCPG (StrornalScore), respectively. (*P* < 0.05).

**Figure 10:**
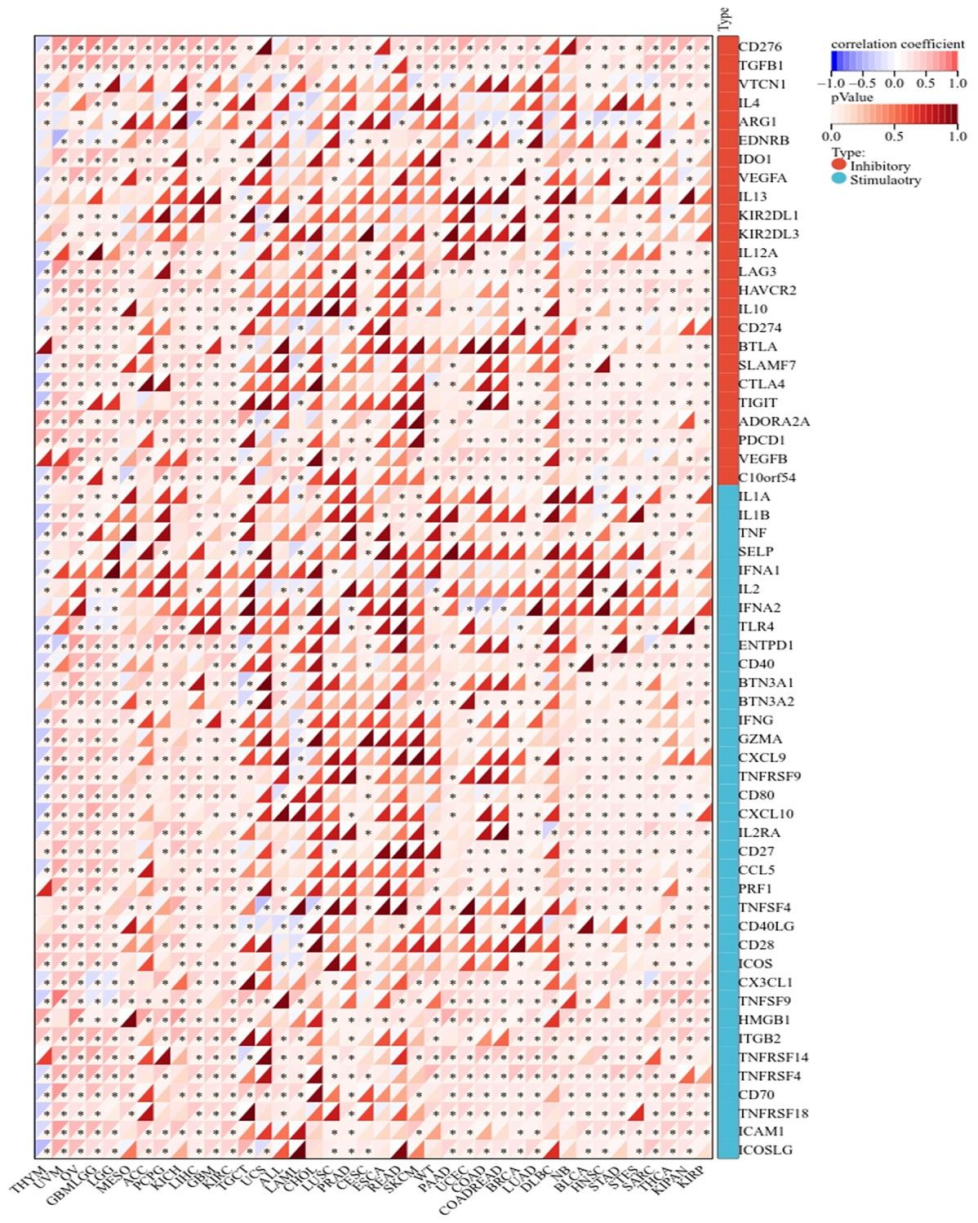
Correlation analysis of BAX gene expression in immune checkpoint.

Immune cells play important roles in regulating tumor development [31]. To reveal the role of BAX in the pan-cancer immune microenvironment, we evaluated the correlation between BAX expression and six types of immune cells by TISIDB, aided by six other algorithms (Figure 8A and Supplementary Figure 4). We observed that BAX expression was positively correlated with the degree of immune infiltration in a variety of cancers. And there is a significant positive correlation in KIRC, KIPAN, LGG, LIHC and THYM particularly (r > 0, *p* < 0.05). Three tumors associated with prognosis, KIRC, LGG and LIHC were screened out for further verification via TIMER algorithm (r > 0, *P* < 0.05), and the results were consistent across two algorithms (Figure 8B). Furthermore, we assessed the correlation between BAX expression and 28 types of TILs in human cancers. The heat map showed that THCA, LGG and SARC were positively correlated with almost all TILs (*P* < 0.05) (Figure 9A). As shown in Supplementary Figure 5, we showed scatter plots of these three cancers. We also explored the tumor immune score of BAX (Figure 9B). Moreover, in defensive response, the tumor could quickly evolve by upregulating immune checkpoint ligands to educate immune cells [32]. Given the large number of reported associations between genes and immune response checkpoint, our studies showed that the immune infiltration-related cancers (THYM, LGG, GBMLGG, LIHC, KIRC, SARC and THCA) were highly positively correlated with many immune checkpoints. The stimulatory checkpoints are ENTPD1, TNFRSF9, CD80, IL2RA, CD27, CD28, CX3CL1, TNFSF9, ITGB2, TNFRSF4, CD70, ICAMI and ICOSLG (r > 0, *p* < 0.05). And inhibitory checkpoints are CD276, TGFB1, LAG3, HAVCR2 and SLAMF7 (r > 0, *p* < 0.05) (Figure 10 and Supplementary Table 2).

### Analysis of BAX methylation

**Figure 11:**
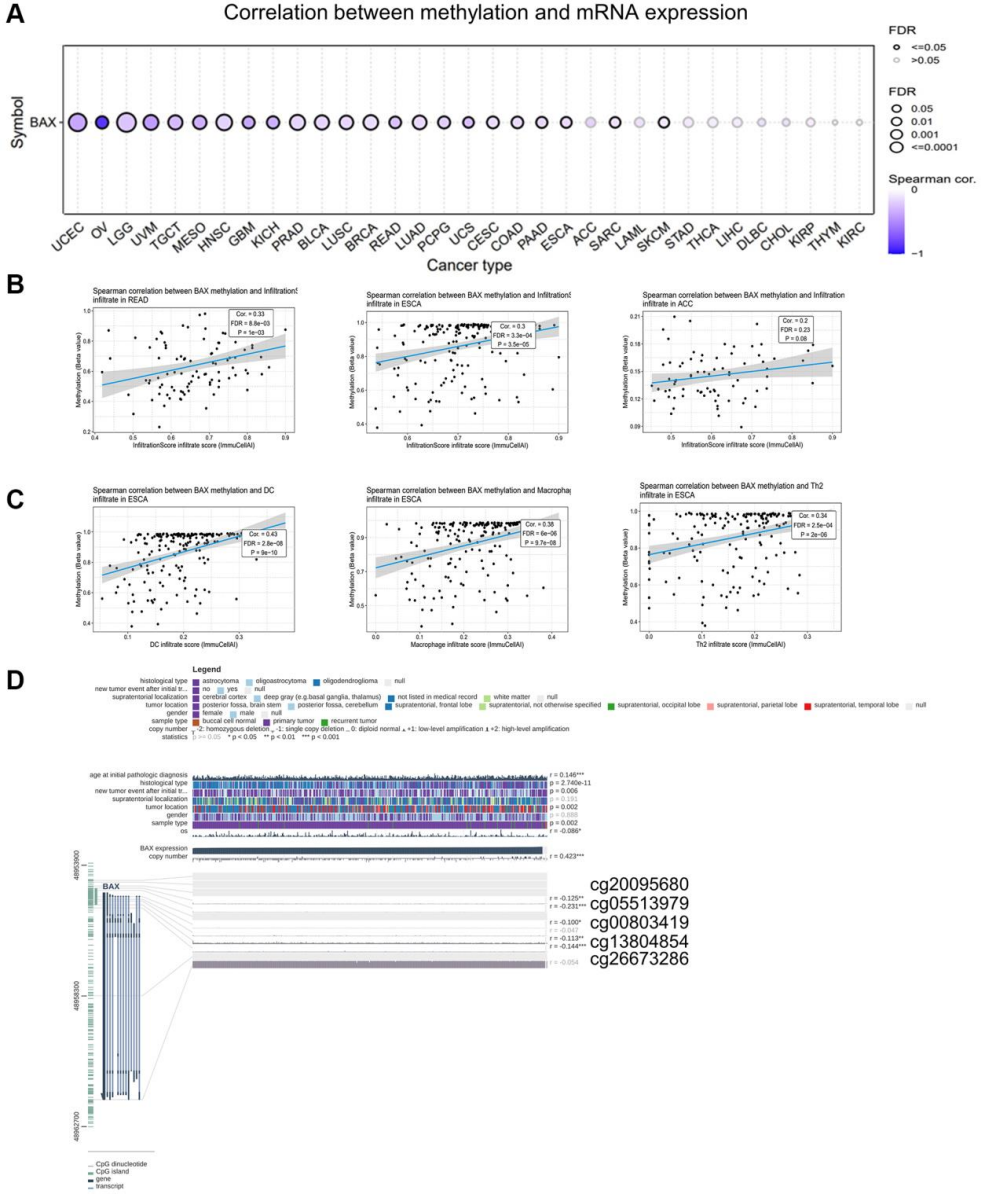
**Analysis of BAX methylation in pan-cancer.** (**A**) A Spearman association between BAX methylation and mRNA expression was performed in pan-cancer. (**B**) The top three with the highest correlation between BAX methylation and immune infiltration. (*P* < 0.05). (**C**) The top three with the highest correlation between BAX methylation and immune cells in ESCA. (*P* < 0.05). (**D**) The methylation sites of BAX DNA sequence related to its gene expression.

GSCA online tool provides an opportunity to analyze the BAX methylation. We figured out that BAX methylation showed a strong link with BAX mRNA expression in most cancer types, such as UCEC, LGG (FDR < 0.0001), UVM, HNSC, PRAD, LUAD (FDR ≤ 0.001), OV, TGCT, MESO, GBM, KICH, BLCA, BRCA, READ, PCPG, USC, CESC, COAD, PAAD, ESCA, SARC and SKCM (FDR < 0.05) (Figure 11A). Especially in OV, the relevance is particularly evident. In addition, we investigated the relationship between BAX methylation and immune infiltration. Figure 11B showed the top three with the highest correlation scores, which were READ, ESCA and ACC. Interestingly, ESCA showed the highest and significant correlation value of TILs (Figure 11C). Besides, we found five methylation sites (cg20095680, cg05513979, cg00803419, cg13804854, cg26673286) in the DNA sequences of BAX is significantly correlated with the expression of BAX (Figure 11D).

### Proliferation and metastasis

**Figure 12:**
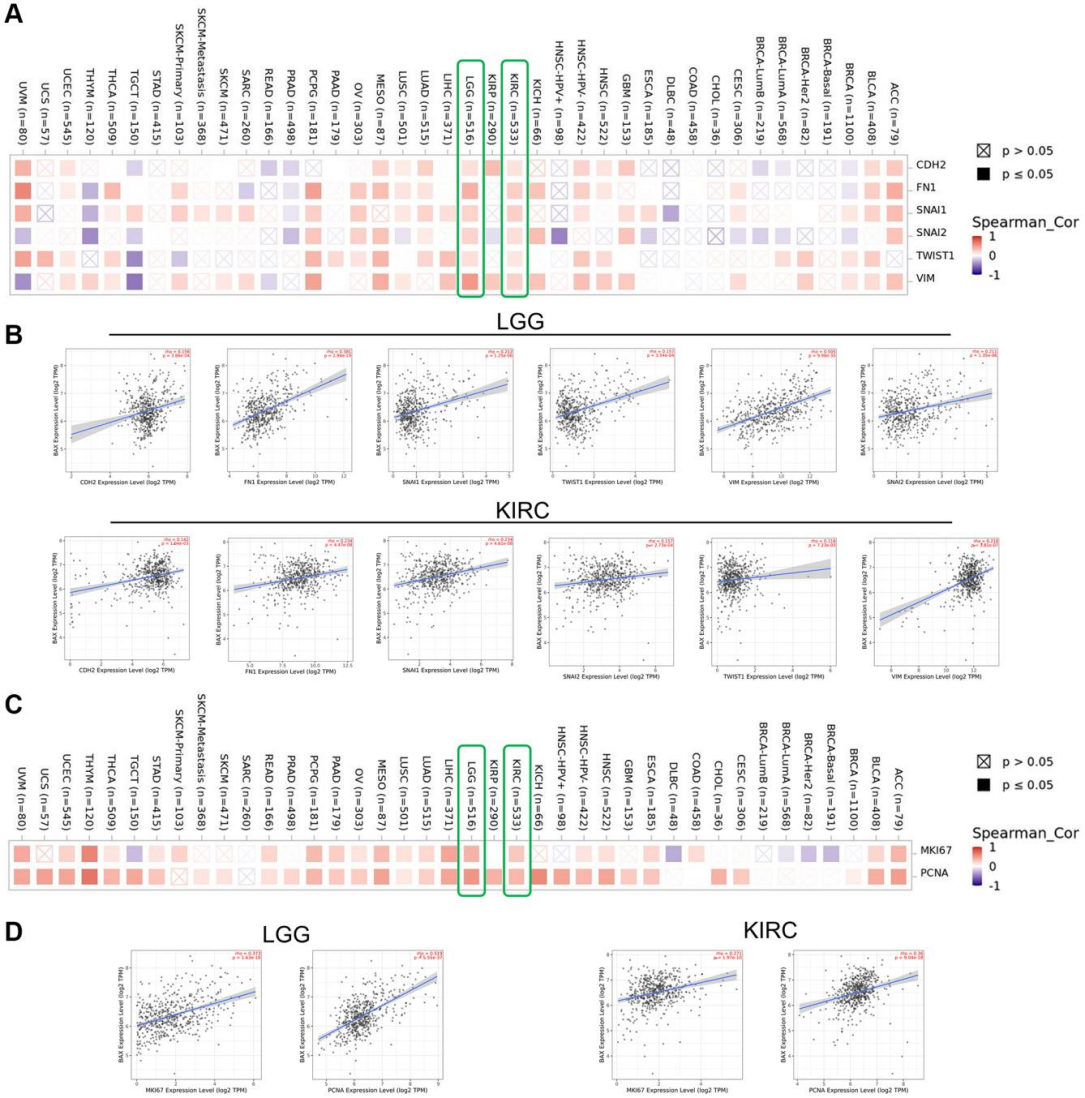
**Correction analysis between BAX and cell proliferation and EMT.** (**A**, **B**) Correlation analysis on the association between BAX expression and EMT markers (CDH2, Fibronectin 1, Snail1, Snail2, TWIST1 and Vimentin), especially in LGG and KIRC. (**C**, **D**) Associations between BAX expression level and proliferation markers (MKI67 and PCNA) were investigated in different cancer types, especially in LGG and KIRC.

Various biological processes such as proliferation and metastasis could play key roles on the occurrence and development of cancers. We focused on the potential associations between BAX and the epithelial–mesenchymal transition (EMT) markers, CDH2 (N-cadherin), FN1 (Fibronectin 1), SNAI1 (Snail1), SNAI2 (Snail2), TWIST1 (Twist Family BHLH Transcription Factor 1) and Vimentin (VIM). The corresponding figure showed that BAX was positively correlated with the expression of these EMT markers of LGG and KIRC (r > 0, *p* < 0.05) (Figure 12A, 12B). Moreover, we revealed the correlation between BAX and the classic proliferation markers, MKI67 (Marker of Proliferation Ki-67) and PCNA (Proliferating Cell Nuclear Antigen). Results proved that BAX was positively correlated with the expression of these proliferation markers across multifarious types of cancer, such as LGG, KIRC, LICH, ACC, BLCA, ESCA, HNSC, LUAD, LUSC, MESO, OV, PAAD, PCPG, READ, STAD, THCA, THYM, UCEC and UVM (r > 0, *P* < 0.05) (Figure 12C). These results suggested that BAX may participate in proliferation and metastasis of many cancers, especially in LGG and KIRC (Figure 12D).

### Enrichment analysis of BAX

**Figure 13:**
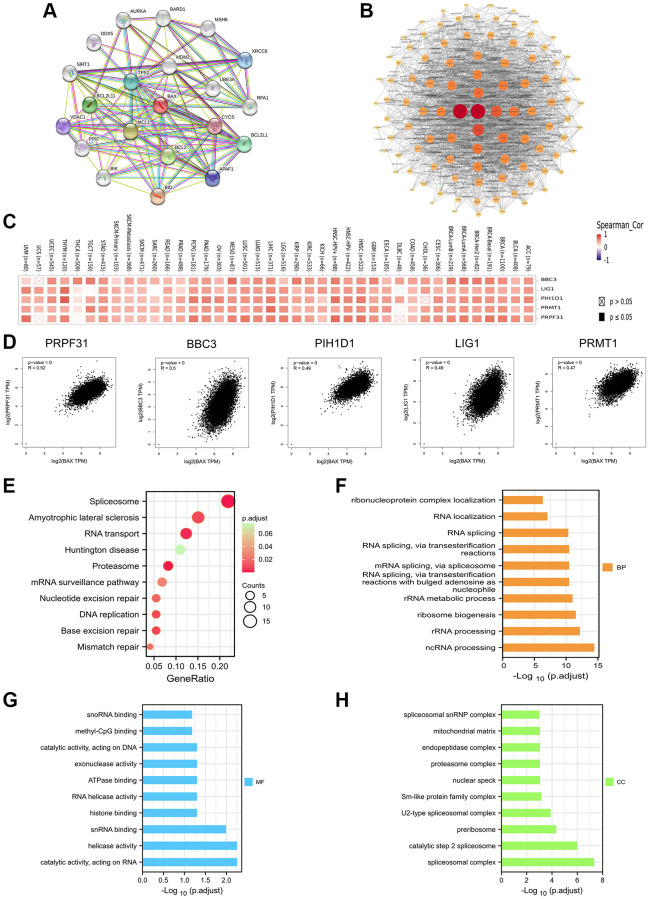
**Enrichment analysis of BAX-related gene.** (**A**) Analysis of twenty proteins interacting with BAX through STRING database. (**B**) Protein–protein interaction network of BAX was performed via the STRING database. (**C**) The heat map of correlation between BAX and the top 5 genes (PRPF31, BBC3, PIH1D1, LIG1 and PRMT1) in TCGA tumors. (**D**) Correlation of BAX and the top 5 genes in the cancer types of TCGA. (**E**) Bubble plot for KEGG pathway analysis. (**F**–**H**) GO enrichment analysis of the top 100 genes that associated with BAX expression.

We profiled twenty proteins interacting with BAX through STRING database and constructed the protein–protein interaction (PPI) network (Figure 13A). Subsequently, to explore this relationship in depth, we concluded 100 proteins on the Cytoscape software. The visual diagram proved that it has the strongest correlation with TP53 (Tumor Protein P53), followed by MDM2 (MDM2 Proto-Oncogene), AKT1 (AKT Serine/Threonine Kinase 1) and ATM (ATM Serine/ Threonine Kinase) (Figure 13B). Furthermore, we used GEPIA2 tool to collect the expression data across all TCGA cancers and got the top 200 genes that most correlated with BAX expression, and the top 5 genes are PRPF31 (Pre-MRNA Processing Factor 31, R = 0.52), BBC3 (BCL2 Binding Component 3, R = 0.5), PIH1D1 (PIH1 Domain Containing 1, R = 0.49), LIG1 (DNA Ligase 1, R = 0.49) and PRMT1 (Protein Arginine Methyltransferase 1, R = 0.47). We used the TIMER2.0 tool to create heat maps and found a strong positive correlation between BAX and the first five genes in most cancer types (Figure 13C, 13D and Supplementary Table 3). Interestingly, many literatures have reported that these five genes are associated with a variety of cancers, such as ovarian cancer, gastric cancer and pancreatic cancer. And lower-grade glioma and liver cancer are related to the prognosis of BAX as well [33–38]. To further investigate the possible function of BAX, we performed GO and KEGG pathway enrichment analysis of the top 200 correlated genes of BAX. The enrichment of KEGG correlated with BAX is the spliceosome, amyotrophic lateral sclerosis and RNA transport (*P* < 0.05) (Figure 13E). Besides, we divide the enrichment of GO into three subjects, CC (cellular component), BP (biological process) and MF (molecular function). The analysis of BP mainly involved in ncRNA processing, rRNA processing and ribosome biogenesis (Figure 13F). The analysis of MF was related to catalytic activity, acting on RNA, helicase activity and snRNA binding (Figure 13G). And the analysis of CC refers to spliceosomal complex, catalytic step 2 spliceosome and preribosome (Figure 13H). These results suggested the possible molecular mechanism of BAX in cancer pathogenesis.

## DISCUSSION

At present, the prognosis and immune infiltration of BAX is largely unexplored and lacks systemic assessment among 33 human cancers. Herein, we performed a comprehensive analysis to explore that BAX gene plays an important role in the development of many cancers. Apoptosis is a hallmark of multifarious human cancers and often correlated with worse prognosis [39]. And BAX participates in a critical step of the mitochondria-dependent apoptosis, which is frequently dysregulated in cancers [40]. Unexpectedly, accumulating evidence suggested that apoptosis is one of the important components of human immunity. The expression of BAX also regulates the sensitivity of cancer cells toward the immune system [41]. Therefore, we aim to reveal the impact of this potential prognostic biomarker on cancer progression and immunotherapy efficacy.

Thus, in this study we have evaluated the relationship between BAX gene and protein expression, survival prognosis, clinical relevance, genetic alteration, Immune infiltration, methylation level and gene enrichment analysis across 33 TCGA cancer types. We found that BAX expression is up-regulated in most cancers. Previous studies have indicated that BAX is a proto-oncogene [42, 43]. The same proto-oncogene showed different expression in different cancers, which is related to the complex interaction between Bcl-2 family members. BAX interacted with other molecules and maintain balance, so as to play their biological function better [44, 45]. We further investigated the expression differences of BAX protein in multifarious cancer types, and BAX expression was significantly elevated in many tissues, including GBM, LIHC, LUAD and COAD. The analysis results of RNA and protein expression levels were inconsistent, this discrepancy may be due to the RNA post-translational modifications, such as ubiquitination, phosphorylation and methylation. In order to further verify the expression of BAX in tumor tissues, we downloaded immunohistochemical staining of these cancers.

In LGG, SKCM, LIHC, GBM, MESO and UVM, higher expression of BAX was associated with poorer OS. And high BAX expression was linked with poor DFS in LGG, SKCM, ACC, LUSC and PRAD. In the above-mentioned 9 cancer types, the expression of BAX is associated with poor prognosis. Combined with clinical assessments, BAX might act as a prognosis biomarker. These results go along with many previous studies [46, 47]. Interestingly, in COAD and UCEC, BAX expression was slightly correlated with better prognosis.

In terms of more clinical relevance, we indicated that the expression of BAX has significant difference in cancer stages. Moreover, the ROC curve showed an excellent performance that there are 16 cancers with an AUC value greater than 0.8. Importantly, by studying the EMT markers and classic proliferation markers, we figured out that BAX may participate in proliferation and metastasis of many cancers, especially in LGG and KIRC. It is worth noting that the mRNA expression of BAX is strongly associated with drug sensitivity of many drugs, especially nutlin-3. In addition, Kale et al. also confirmed that phosphorylation switches the BAX function from pro- to anti-apoptotic, and thereby increasing drug resistance [48].

Genetic and epigenetic alterations have been found in every region of the protein virtually in cancer progression but only a handful of the mutation sites have been studied in depth. The mutation of BAX gene was mainly missense mutation, and the mutation frequency of R89Q was the highest. Besides, R89Q mutations have also been reported in several mild patients with mucopolysaccharidosis type I (MPS I). The relationship between BAX and CNV was also studied in depth. We observed that in LGG, BAX CNV has a significant positive correlation with clinical survival.

The tumor microenvironment (TME) is highly associated to the cancer development, which including abundant infiltrating immune cells. We suggested that BAX expression was positively correlated with six types of immune cells in LGG, LIHC and KIRC. TILs in the TME have been proven as potential prognostic and therapeutic biomarkers in clinical practice [49]. Our study investigated that BAX expression was positively correlated with almost all TILs in LGG, THCA and SARC. Immune checkpoint therapy has shown considerable promise in treatment of cancers currently, many immune checkpoints are highly positively correlated with BAX, such as ENTPD1, CD80, and CD28. ENTPD1, otherwise known as CD39, which is distributes on various cells in the TME. It could also be the main extracellular enzyme catabolizing ATP in TME [50, 51]. Due to the broad immune regulatory effects on the tumor immunity cycle, targeting CD39 is one of the most promising approaches in immuno-oncology [52]. Subsequently, CD80 is an additional binding partner of PD-L1. There is an inhibitory bidirectional interaction between CD80 and PD-L1, and the CD80 family may regulate T cell activation and tolerance [53]. Meanwhile, CD28 is also a primary target for PD-1-mediated inhibition. It plays a key role in regulating effector T cell function and responses to anti-PD-L1/PD-1 therapy [54]. The above findings proved the potential immune function of BAX in LGG by combining with immune scores. For the first time, we revealed the remarkable relationship between BAX methylation and cancers, and BAX methylation level could be used as a biomarker for the prognosis.

By summarizing more than 100 interacting proteins, PPI network showed a demonstration that BAX has the strongest correlation with TP53, and the gene is the most commonly mutated gene across 33 TCGA cancer types [55]. Next, the results of GO and KEGG enrichment analysis predicted the vital role of BAX in RNA processing and RNA transport. These results displayed that BAX may be involved in a variety of biological processes.

Interestingly, we found that BAX was associated with poor prognosis of LGG, and was also involved in its proliferation and migration. BAX not only showed significant clinical relevance, but also has potential immune function in LGG. Our study undoubtedly provided a new guidance for the treatment of LGG. However, even though our research is based on several databases, there were still some inevitable limitations. The study highlights the importance of investigating the immune response of the BAX gene to tumors in pan-cancer to improve the understanding of the tumor microenvironment. Meanwhile, we predict that BAX is a pan-cancer prognostic biomarker, and targeting BAX may be a viable therapeutic strategy for many cancers to enhance the efficacy of immunotherapy. In the future, we will further study the complex interaction between BAX gene and BCL-2 family members, as well as its potential mechanism in the process of immune infiltration, so as to provide a theoretical basis for the development of immunotherapy. Although our study is based on a large amount of publicly available data in several databases, we are limited by the inevitable limitation that the specific role of BAX in various tumors needs to be confirmed in more extensive clinical cases.

## CONCLUSION

In summary, we provide a comprehensive analysis of BAX for the first time. Our findings demonstrate that BAX was highly expressed in most cancers and the high expression is mostly associated with poor prognosis. In addition, BAX showed significant clinical relevance, meanwhile its expression was positively correlated with most immune infiltrating cells. Importantly, BAX is also known as a pro-apoptotic protein, participated in the process of immune regulation. Meanwhile, the mis-regulation of apoptosis is a hallmark of human cancers and autoimmune disease. These studies indicated that BAX may promote the cancer initiation and progression. It can function as an oncogene and a potential biomarker for prognosis and immunotherapy efficacy of human cancer.

## Supplementary Materials

Supplementary Figures

Supplementary Table 1

Supplementary Table 2

Supplementary Table 3
