# Supplementary material for: Pro-apoptotic gene BAX is a pan-cancer predictive biomarker for prognosis and immunotherapy efficacy
Source: Aging (Albany NY). 2024 Jul 5;16(14):11289–317. doi: 10.18632/aging.206003 (PMC11315380; doi:10.18632/aging.206003)
Supplement: Supplementary Figures [file aging-16-206003-s001.pdf]

SUPPLEMENTARY FIGURES

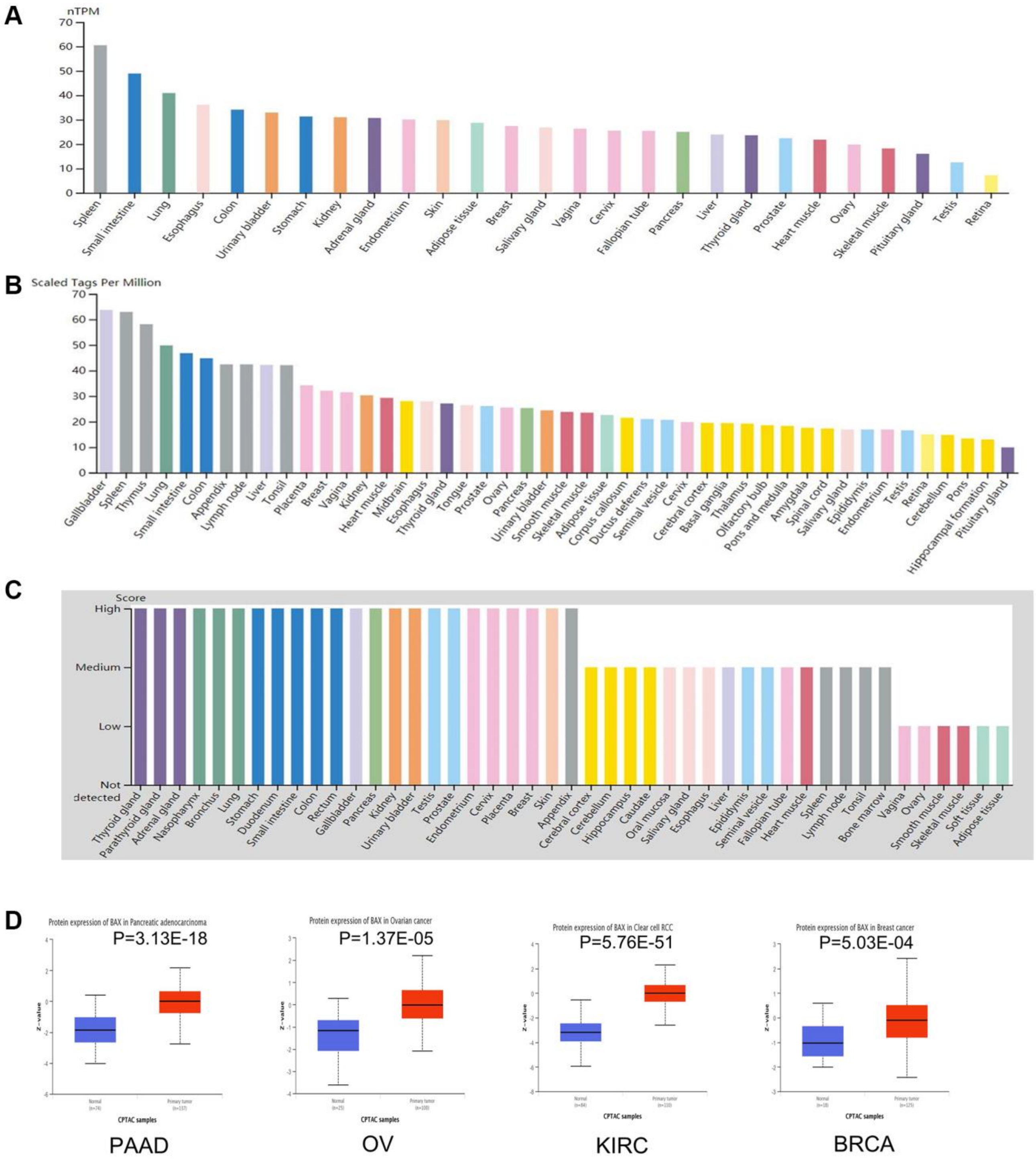

Supplementary Figure 1. (**A**–**D**) Expression level of BAX in different tissues and the protein expression of BAX were greater in some other tumor tissues than in adjacent normal tissues, such as PAAD, OV, KIRC and BRCA.

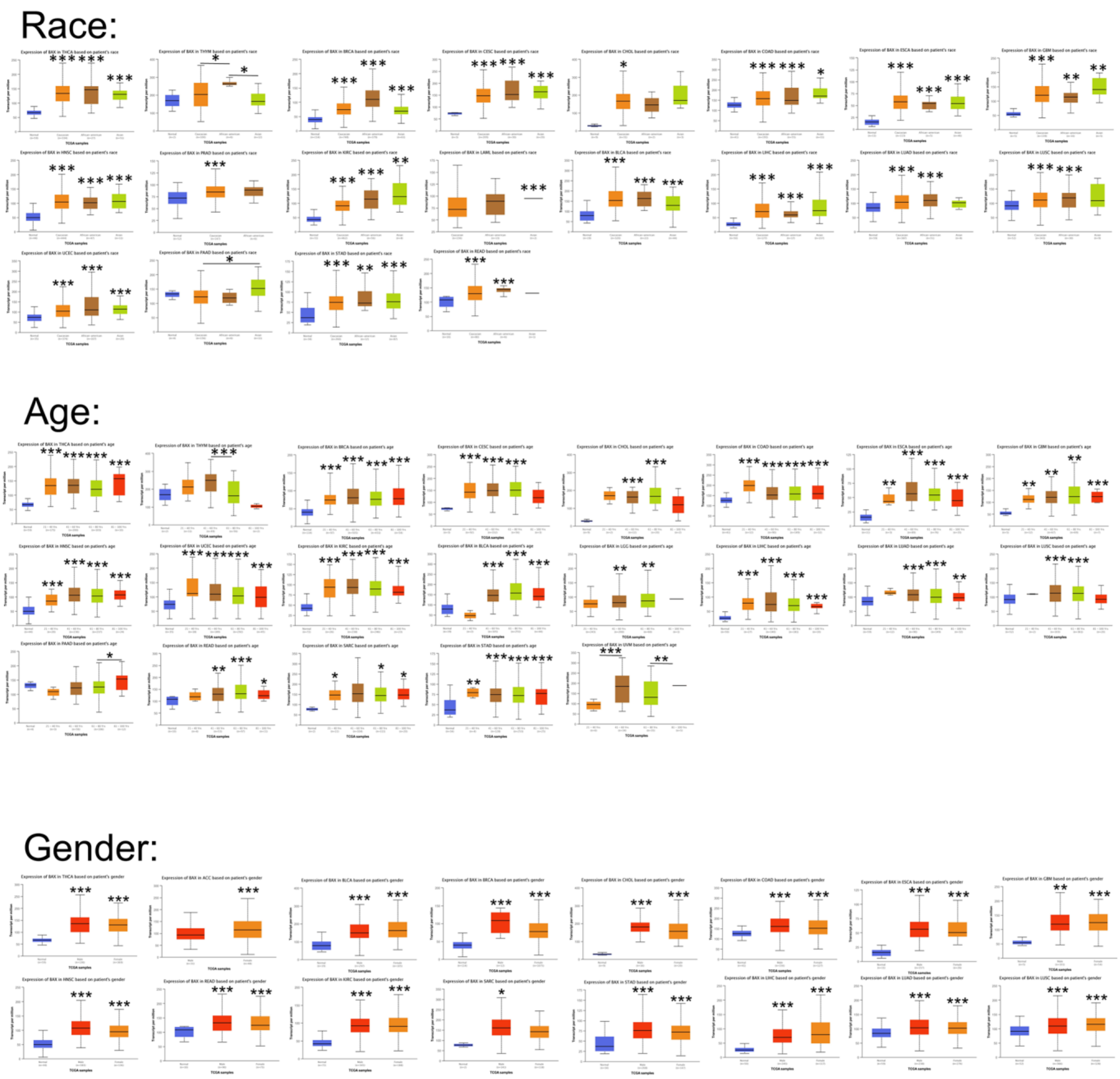

Supplementary Figure 2. BAX expression was significantly associated with age, gender and race in great majority of tumors. ($^*P < 0.05$, $^{**}P < 0.01$, $^{***}P < 0.001$).

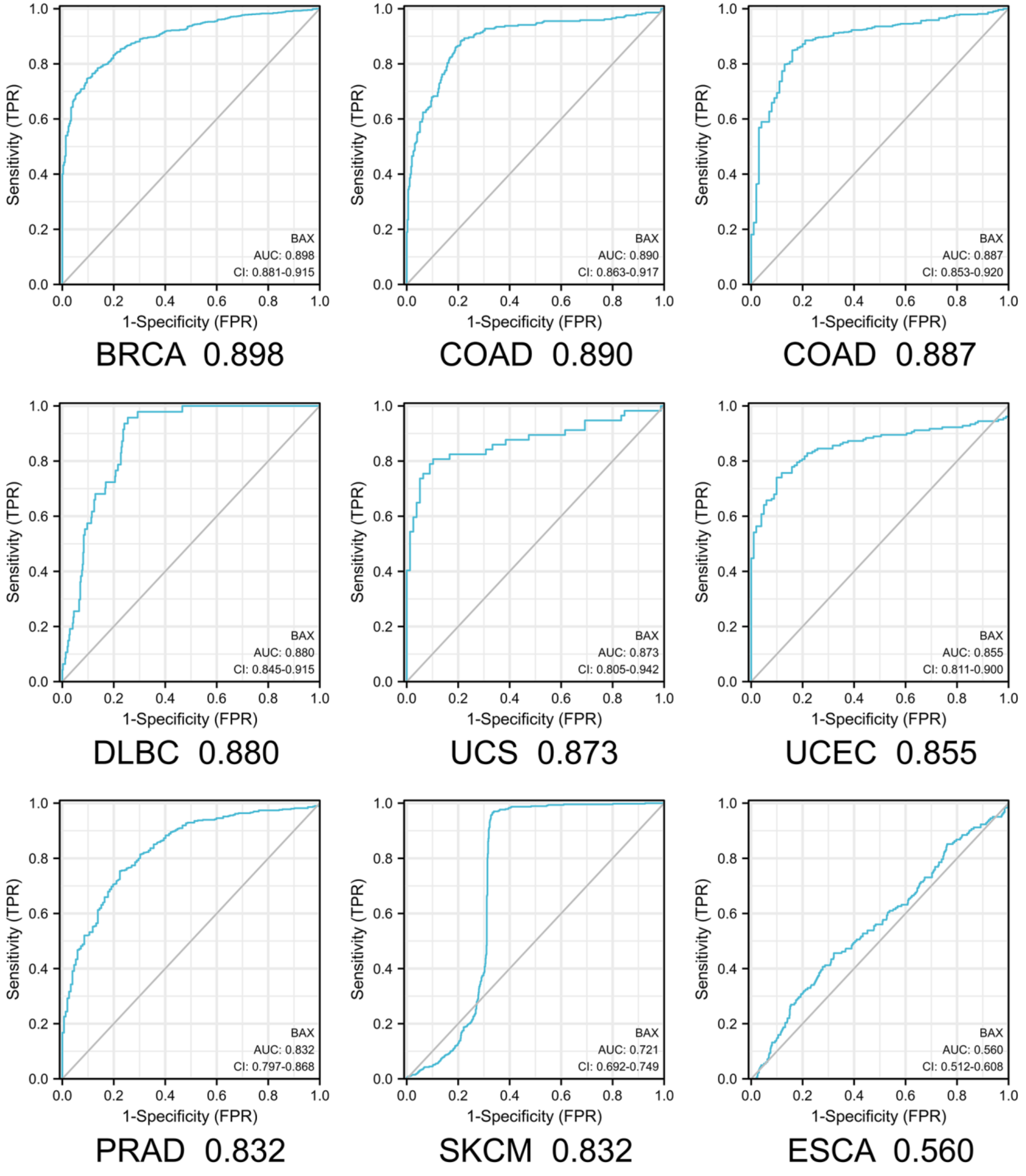

Supplementary Figure 3. The AUC value of ROC curve of BAX from 0.5 to 0.9 for many cancer types.

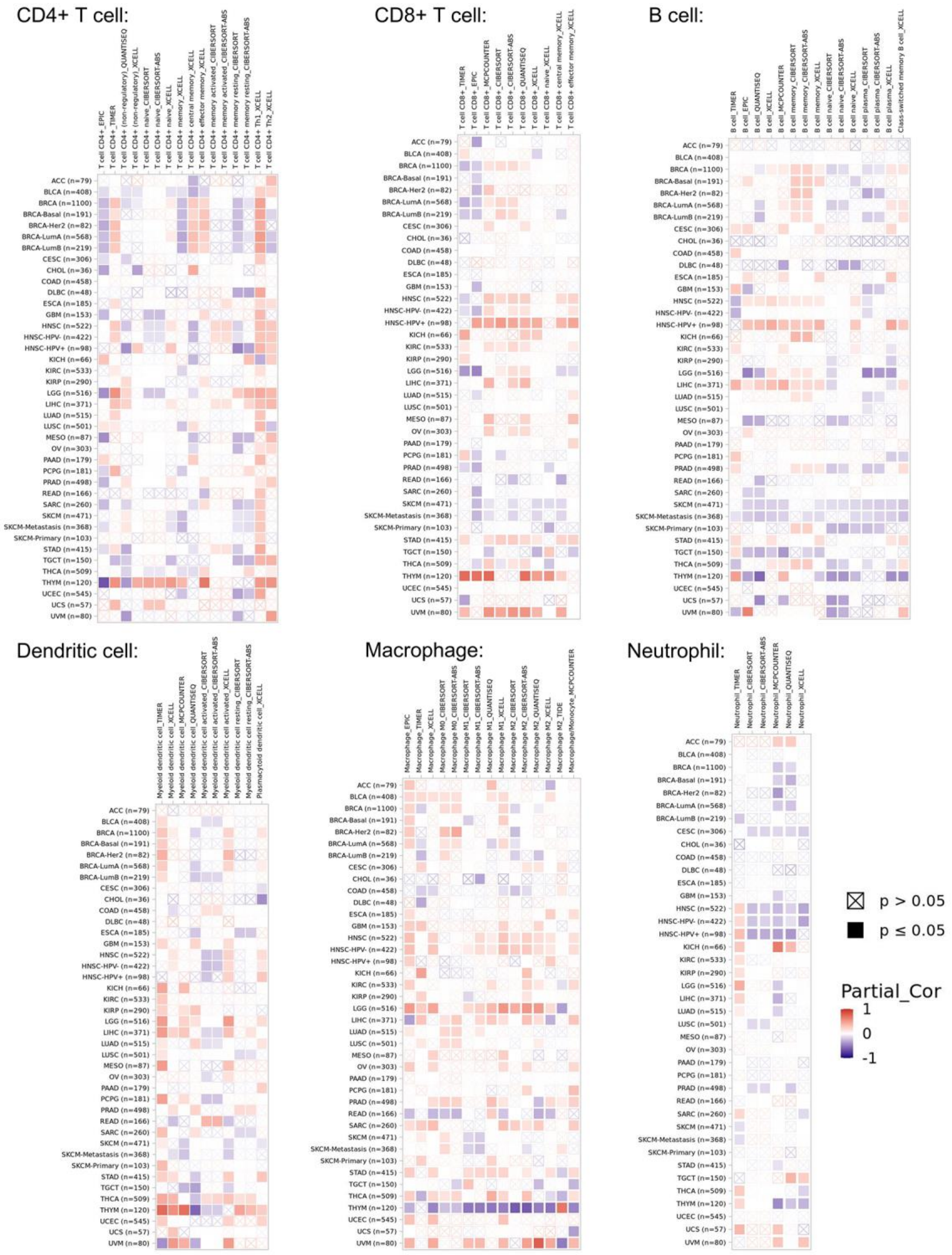

Supplementary Figure 4. The six algorithms suggested that BAX was closely related to immune infiltration of various cancers. ($P < 0.05$).

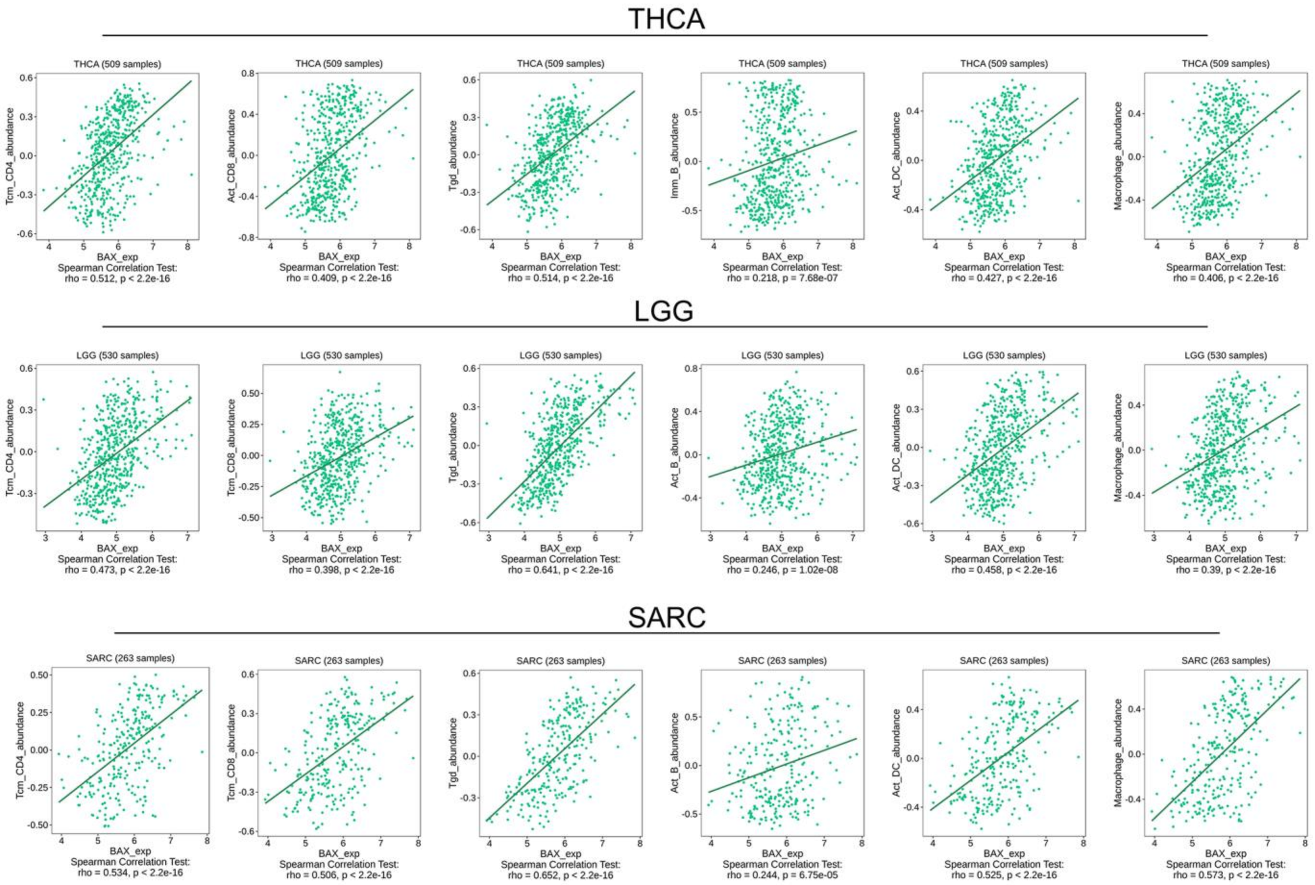

Supplementary Figure 5. The scatter plots of THCA, LGG and SARC were positively correlated with almost all TILs. ($P < 0.05$).
