# Supplementary material for: Pro-apoptotic gene BAX is a pan-cancer predictive biomarker for prognosis and immunotherapy efficacy
Source: Aging (Albany NY). 2024 Jul 5;16(14):11289–317. doi: 10.18632/aging.206003 (PMC11315380; doi:10.18632/aging.206003)
Supplement: Supplementary Table 1 [file aging-16-206003-s002.pdf]

SUPPLEMENTARY TABLE

Supplementary Table 1. Box plot representation of BAX expression level comparison in pan-cancer (TCGA project) relative to the corresponding normal tissues (GTEx database).

Group1	Group2	Number	Min	Max	Median	IQR	Mean	SD	SE
ACC	Normal	128	0	6.963	6.088	0.562	5.95	0.794	0.07
ACC	Tumor	77	3.252	8.108	6.536	0.909	6.519	0.818	0.093
BLCA	Normal	28	5.423	7.604	6.271	0.697	6.291	0.528	0.1
BLCA	Tumor	407	4.535	8.917	7.293	0.742	7.292	0.591	0.029
BRCA	Normal	292	3.291	6.649	5.639	0.607	5.576	0.526	0.031
BRCA	Tumor	1099	3.892	8.203	6.532	0.68	6.507	0.563	0.017
CESC	Normal	13	5.851	6.425	6.061	0.278	6.104	0.182	0.05
CESC	Tumor	306	4.782	8.421	7.342	0.582	7.271	0.471	0.027
CHOL	Normal	9	4.72	5.529	5.022	0.086	5.069	0.231	0.077
CHOL	Tumor	36	5.177	9.011	7.43	0.801	7.418	0.696	0.116
COAD	Normal	349	0	7.804	6.087	0.99	6.097	0.919	0.049
COAD	Tumor	290	3.392	8.879	7.354	0.681	7.275	0.715	0.042
DLBC	Normal	444	1.978	9.138	6.061	2.068	6.175	1.389	0.066
DLBC	Tumor	47	6.135	9.338	8.174	0.687	8.104	0.575	0.084
ESCA	Normal	666	0	7.138	5.746	0.644	5.713	0.698	0.027
ESCA	Tumor	182	4.323	7.134	5.65	0.723	5.671	0.511	0.038
GBM	Normal	1157	0	7.38	4.821	0.903	4.72	0.838	0.025
GBM	Tumor	166	4.848	8.591	7.32	0.74	7.303	0.586	0.045
HNSC	Normal	44	2.807	7.176	5.855	0.554	5.794	0.757	0.114
HNSC	Tumor	520	5.07	8.387	6.786	0.619	6.78	0.496	0.022
KICH	Normal	53	0	7.401	5.954	0.824	5.683	1.118	0.154
KICH	Tumor	66	3.573	7.781	5.663	1.011	5.693	0.71	0.087
KIRC	Normal	100	0	7.143	5.66	0.661	5.561	0.844	0.084
KIRC	Tumor	531	3.599	8.14	6.718	0.703	6.635	0.626	0.027
KIRP	Normal	60	0	6.887	5.692	0.831	5.589	1.019	0.131
KIRP	Tumor	289	5.403	8.506	7.262	0.747	7.188	0.595	0.035
LAML	Normal	70	5.204	6.363	5.971	0.323	5.97	0.246	0.029
LAML	Tumor	173	4.544	8.205	6.032	0.936	6.067	0.639	0.049
LGG	Normal	1152	0	7.38	4.816	0.903	4.716	0.836	0.025
LGG	Tumor	523	4.592	8.423	6.402	0.748	6.474	0.606	0.026
LIHC	Normal	160	2.795	7.344	4.658	0.881	4.753	0.742	0.059
LIHC	Tumor	371	3.638	8.747	6.318	0.987	6.341	0.759	0.039
LUAD	Normal	347	0	8.085	6.654	0.594	6.605	0.632	0.034
LUAD	Tumor	515	3.561	8.247	6.661	0.696	6.623	0.565	0.025
LUSC	Normal	338	0	8.085	6.681	0.588	6.63	0.639	0.035
LUSC	Tumor	498	4.489	8.215	6.846	0.703	6.811	0.506	0.023
MESO	Tumor	87	6.106	8.263	7.216	0.913	7.178	0.597	0.064

OV	Normal	88	4.542	6.417	5.408	0.546	5.436	0.391	0.042
OV	Tumor	427	0	7.388	5.778	0.808	5.747	0.691	0.033
PAAD	Normal	171	0	7.495	4.468	0.721	4.46	0.863	0.066
PAAD	Tumor	179	4.207	8.311	7.06	0.532	7.035	0.546	0.041
PCPG	Normal	3	5.262	6.075	5.533	0.406	5.623	0.414	0.239
PCPG	Tumor	182	4.305	8.082	6.2	0.799	6.228	0.663	0.049
PRAD	Normal	152	5.009	7.007	6.033	0.594	6.032	0.44	0.036
PRAD	Tumor	496	4.615	8.716	6.635	0.538	6.625	0.477	0.021
READ	Normal	318	0	7.804	5.972	0.865	6.002	0.906	0.051
READ	Tumor	93	5.589	8.468	7.087	0.616	7.15	0.521	0.054
SARC	Normal	2	5.944	6.524	6.234	0.29	6.234	0.41	0.29
SARC	Tumor	262	5.34	8.768	7.275	0.734	7.24	0.576	0.036
SKCM	Normal	813	4.481	8.91	5.72	2.413	6.305	1.248	0.044
SKCM	Tumor	469	5.077	9.235	7.325	0.773	7.302	0.611	0.028
STAD	Normal	210	0	7.036	5.377	0.83	5.303	0.816	0.056
STAD	Tumor	414	3.352	8.53	6.054	0.886	6.058	0.712	0.035
TGCT	Normal	165	4.26	6.955	5.286	0.521	5.29	0.485	0.038
TGCT	Tumor	154	4.692	8.854	7.883	0.788	7.744	0.714	0.058
THCA	Normal	338	0	7.186	6.086	0.519	6.085	0.489	0.027
THCA	Tumor	512	5.418	8.744	7.026	0.603	6.993	0.478	0.021
THYM	Normal	446	1.978	9.138	6.068	2.067	6.18	1.388	0.066
THYM	Tumor	119	5.577	8.554	7.714	0.864	7.578	0.607	0.056
UCEC	Normal	101	5.146	7.19	6.183	0.582	6.148	0.417	0.041
UCEC	Tumor	181	3.752	8.892	7.083	0.965	7.016	0.859	0.064
UCS	Normal	78	5.146	7.19	6.085	0.547	6.117	0.417	0.047
UCS	Tumor	57	5.36	8.338	7.153	0.717	7.021	0.624	0.083
UVM	Tumor	79	4.894	8.344	7.153	1.311	7.065	0.769	0.087
